# Economy in the Annual Report

**Published:** 1916-06-17

**Authors:** 


*To the Editor of* The Hospital.

### Economy in the Annual Report.

*To the Editor of* The Hospital.

Sir,—I am sorry my letter in your issue of May 27 should have been construed as deprecating saving in printing and circulating the annual report. This was not my intention. What I wished to deprecate was the statement that between £200 and £300 can be saved by the means quoted. If a saving can be effected by omitting extraneous matter, by all means let us have it, but if the report has been economically printed and circulated it would not appear possible to save the amount mentioned. May I join with Mr. Ryan in asking Charing Cross Hospital to tell us how it is done?—I have the honour to be, your obedient servant,

June 10, 1916. Assistant Secretary.

